# A Practical Guide to Using Futures Methods in Health Care: Approaches, Applications, and Case Studies

**DOI:** 10.2196/82820

**Published:** 2025-09-16

**Authors:** Bertalan Mesko, Tamás Kristóf, Pranavsingh Dhunnoo, Nóra Árvai, Gellért Katonai

**Affiliations:** 1 The Medical Futurist Institute Budapest Hungary; 2 Kálmán Laki Doctoral School of Biomedical and Clinical Sciences University of Debrecen Debrecen Hungary; 3 Institute of Entrepreneurship and Innovation Corvinus University of Budapest Budapest Hungary; 4 Department of Computing Atlantic Technological University Letterkenny Ireland; 5 Meducation Hungary Kft Budapest Hungary; 6 Povl Bang-Jensen u. 2/B1. The Medical Futurist Institute Budapest Hungary; 7 Department of Family Medicine Semmelweis University Budapest Hungary; 8 Povl Bang-Jensen u. 2/B1. 4/1. The Medical Futurist Institute Budapest Hungary

**Keywords:** health care, future, futures methods, medicine, foresight

## Abstract

Researchers and health care institutions have increasingly applied structured futures methods—such as the futures wheel, scenario analysis, forecasting, and horizon scanning—to systematically explore, generate, and prepare for multiple possible futures. However, discussions around the future of medicine, specialties, or therapeutic areas have often relied on the subjective opinions or perspectives of key opinion leaders rather than on future strategies, policies, visions, and scenarios that are grounded in rigorous and established methods. This underscores the need for futures methods to be widely adopted and effectively incorporated into both medical practice and health care policymaking. Integrating structured foresight techniques into strategic planning enables clinicians and policymakers to transition from reactive decision-making to proactive, plausible approaches that shape a more resilient and adaptive health care system. Our goal with this paper is to provide a methodological guide that is supported by case studies, demonstrating how futures methods can be systematically applied in health care. By offering practical examples, we intend to empower medical professionals, health care leaders, researchers, patients, and policymakers with the tools to anticipate and navigate future challenges and opportunities more effectively.

## Introduction

The paradigm shift of digital health has transformed health care delivery, medical practice, and the roles of patients and health care professionals, as well as the cultural dimensions of care in the 21st century, prompting increased discussions about the future. Unprecedented advancements in technologies, from wearable sensors to artificial intelligence (AI), are directing our attention toward emerging challenges and opportunities. With the accelerating rise of AI and automation, the next paradigm shift of health care is imminent: the inclusion of a technological entity as an active participant within the medical team, alongside patients and health care professionals. The COVID-19 pandemic, a recent focus on value-based health care, and global demographic issues from doctor shortages to aging populations further reinforce that notion.

To better understand these challenges and opportunities, researchers and health care institutions have increasingly applied structured futures methods—such as the futures wheel (FW), scenario analysis (SA), forecasting, and horizon scanning (HS)—to systematically explore, generate, and prepare for multiple possible futures [1]. Such methods have been used in social sciences, economics, and business management for decades. Therefore, while the integration of such methods in health care is not new, it has never been more opportune than today.

However, discussions around the future of medicine, specialties, or therapeutic areas have often relied on the subjective opinions or perspectives of key opinion leaders rather than on future strategies, policies, visions, and scenarios that are grounded in rigorous and established methods.

This underscores the need for futures methods to be widely adopted and effectively incorporated into both medical practice and health care policymaking. Integrating structured foresight techniques into strategic planning enables clinicians and policymakers to transition from reactive decision-making to proactive, plausible approaches that shape a more resilient and adaptive health care system.

Our goal with this paper is to provide a methodological guide that is supported by case studies, demonstrating how futures methods can be systematically applied in health care. By offering practical examples, we intend to empower medical professionals, health care leaders, researchers, patients, and policymakers with the tools to anticipate and navigate future challenges and opportunities more effectively. Textbox 1 provides a brief overview of futures methods.

**A brief overview of futures methods with their key principles.**

The preferred future cannot be attained only by extrapolating existing tendencies. Futures methods seek to systematically investigate, generate, and evaluate possible, probable, preferred, and plausible futures to enhance informed decision-making. These methods encompass the examination of prospective changes in conditions resulting from the implementation of alternative policies and measures, together with the anticipated repercussions of specific policies and measures.

It is contended that the results of initiatives using futures methods significantly rely on the techniques used and the competencies of the participants. Futures methods include both qualitative and quantitative components. It is also feasible to differentiate between exploratory and normative approaches.

The application of futures methods substantially enhances the generation and implementation of futures literacy, hence facilitating prompt action for people or organizations to navigate change more adeptly. The capacity for foresight provides more time to comprehend challenges and possibilities, formulate innovative strategies, design new products, and articulate a vision for prospective organizational transformations.

## Search Strategy

Futures methods that our benchmark study already identified were chosen as the focus of this paper, and we looked for case studies and papers highlighting their practical use. The methods include the FW, SA and scenario planning (SP), backcasting, technology assessment, policy analysis, HS, and the Delphi method.

We performed an exploratory literature search on PubMed and Crossref with search terms derived from previously selected methods (Textbox 2).

**Search terms for PubMed and Crossref.**

“the Futures Wheel” OR “Future Wheel”

“scenario analysis” [Title/Abstract]) AND (future [Title/Abstract]

“scenario planning” [Title/Abstract]) AND (future [Title/Abstract]

“backcasting” [Title/Abstract])

“technology assessment” [Title/Abstract]) AND (future [Title/Abstract]

“policy analysis” [Title/Abstract]) AND (future [Title/Abstract]

“horizon scanning” [Title/Abstract])

“Delphi” [Title/Abstract]) AND (future [Title/Abstract]

We used PubMed to capture health-focused applications of futures methods and Crossref to identify broader interdisciplinary uses across policy, planning, and technology fields, ensuring a comprehensive view on how these methods might have been applied in the health care setting.

Articles were included if they (1) were original research articles, (2) used the specified futures methods, (3) were relevant to medicine or health care, and (4) had at least an English abstract available.

## Futures Methods

### The FW

The FW method helps explore the direct and indirect consequences of an event, milestone, statement, or trend. It was first used by Jerome C Glenn in the 1970s [2]. Creating an FW starts with placing a central event in the middle of a diagram and then mapping out its first-order effects, which will lead to possible second- and third-order consequences. After each primary effect, secondary effects create further circular patterns. To identify secondary impacts, participants are asked to ignore the initial element placed in the center of the FW and instead provide the most likely future outcomes of the primary impacts. Participants can similarly outline third- and fourth-order effects. Several research groups have used this method to map out the consequences of health care and medical events.

Similar to structured brainstorming, FWs are organized around future-related issues and conceptualized within this framework [3]. The impact mechanism persists until the consequences of the event or trend become apparent. To identify future trends and events, futurists ask participants what next steps they expect, what the essential components of the events and trends are, and what impacts or consequences they expect. This creates a cognitive framework for the future, encouraging novel concepts [4].

**Figure 1:**
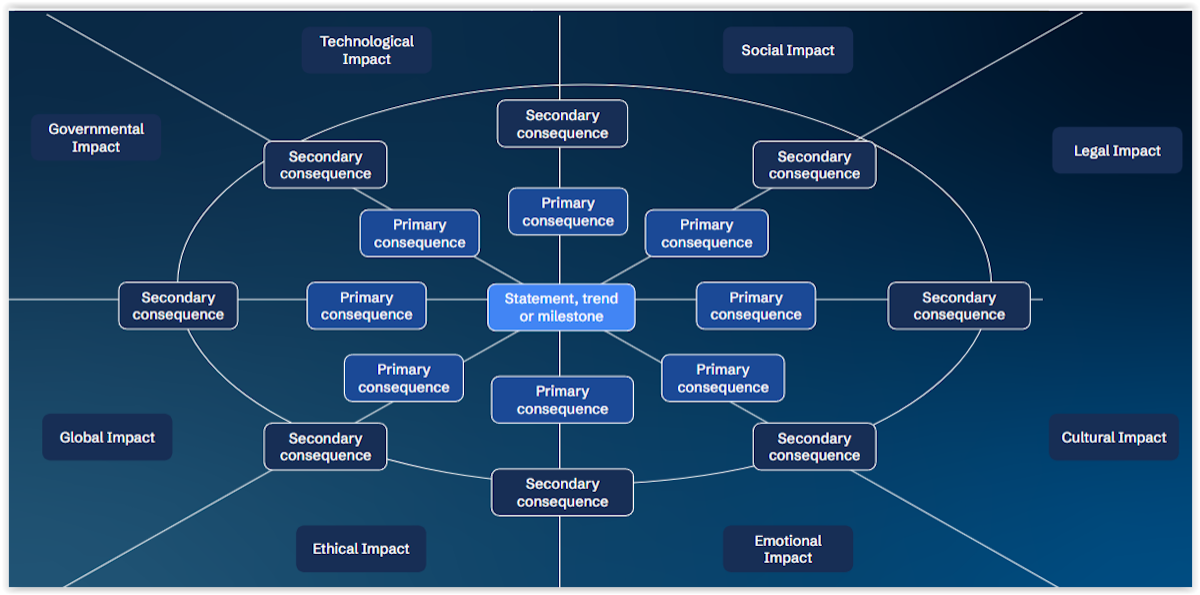
A sample futures wheel with a central statement or trend and primary, then secondary consequences in categories such as legal, technological, or social impact.

FWs are an accessible option that demand neither special software nor specialized equipment. A sheet of paper and a pen, or a schematic diagram, are sufficient to apply the method. We provide a sample diagram in Figure 1. FW variations generally entail alterations in the organization of consequences, such as using categories (eg, economic or technological) or adjusting the visual representation (eg, varying line thicknesses) to facilitate a more nuanced or concentrated analysis.

FWs have been demonstrated to encourage subsequent investigation and comprehension of occurrences and trends. When futurists encounter obstacles in strategic planning, they frequently use FWs to rejuvenate the group’s cognitive flow. The outcomes of FWs can facilitate concept formation, methodical investigation of futures, structuring of future knowledge, and future experimentation [5].

Nouhi et al [6] applied this method to assess the primary and long-term effects of COVID-19 on the health care system. They focused on key dimensions such as service delivery, the workforce, infrastructure, governance, the pharmaceutical industry, research, and medical education. The research started with a systematic literature review, then proceeded with expert panel discussions, and finally concluded with structured brainstorming sessions. This approach enabled the project managers to understand the complexity of COVID-19’s impact and offer systematic foresights into the pandemic’s long-term implications.

Nejatzadehgan Eidgahi et al [7] aimed to discover the impact of pandemics on older populations in Iran. Considering Iran’s demographic shift toward an aging society, comprehending the multifaceted repercussions of pandemics on older individuals is important. The research was conducted in 3 phases. Initially, a panel of geriatric health experts identified a range of pandemic-related effects through brainstorming and literature review. These impacts were further analyzed using the social, technological, economic, environmental, and political (STEEP) framework, classifying them into 6 dimensions: sociopsychological, technological, economic, environmental, political, and health-related consequences. The final consensus phase involved validating the findings and refining the FW model to depict interrelated implications. The research identified 81 distinct effects of infectious disease outbreaks on older people, with 10 categorized as opportunities and 9 categorized as threats. The study concluded that it is necessary to strengthen digital literacy initiatives, broaden mental health support services, and cultivate resilient health care systems tailored to the specific needs of aging populations. The FW provided an essential analytical framework for anticipating future crises.

### SA and SP

**Figure 2:**
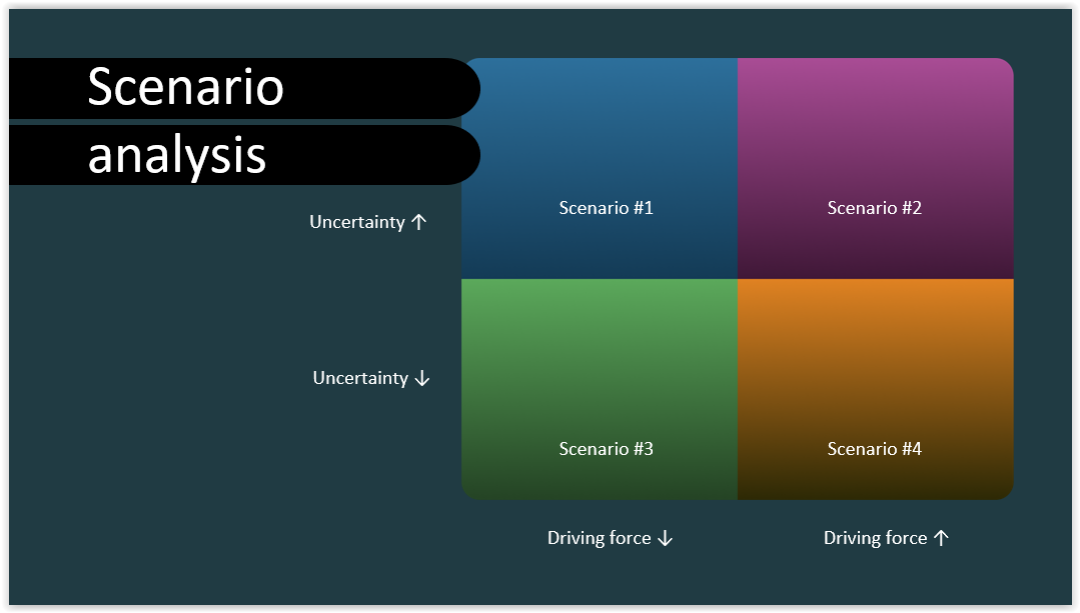
A sample scenario analysis with a key driving force, a key uncertainty, and 4 possible scenarios based on the combinations of the 2 factors.

SA and SP are complementary methods used in foresight. SA helps to discover possible future scenarios based on key driving forces and major uncertainties. SP is a more structured approach, usually used by businesses and governments to prepare for uncertain futures by identifying strategies that can work across different possible circumstances [8]. The primary goal of SP is to find the best combination of strategic actions that might optimize organizational performance amid diverse uncertainties. Futurists point out that the future is never singular, as there are always multiple possible scenarios. SP encompasses a perplexing array of methods. Some authors differentiate between qualitative and quantitative techniques for scenario development, while others prefer to distinguish between prospective and predictive types, often integrating them with more complex methods, such as those based on AI [9]. Foresight methods like SA and SP offer organizations valuable tools to better prepare for a range of possible futures. The next section outlines several examples of how these approaches have been applied in practice (Figure 2).

Leufkens et al [10] demonstrated that SA was already used in medicine as early as 1994. The author aimed to explore how SA can serve as a strategic tool for anticipating the changing dynamics of health care policy. The research involved several steps. It began with a delineation of the industry, followed by identification of critical uncertainties and key driving forces, such as demographic shifts and scientific advancements. These were then categorized into 2 groups, named market regulation and technological trust. Researchers constructed 4 narrative-based scenarios based on quantitative estimations that were obtained from drug consumption databases and demographic forecasts. This mixed-approach study illustrates how qualitative and quantitative methods can be combined.

York et al [11] used SA to examine the future of precision medicine. The study sought to establish a collaborative foresight process that involved both undergraduate students and experts. The methodological framework combined SA with designing fiction, mixing components of storytelling and visual media. Structured workshops adhered to a 3-phase process. Initially, experts and students identified the key drivers that are shaping precision medicine. Secondly, SA was used to detect the varying levels of technological adoption and regulatory policies. Ultimately, participants developed visual and narrative depictions of possible futures. The study demonstrated that scenario-based storytelling can foster interdisciplinary dialogue, and it also highlighted the importance of governance in shaping ethical and equitable medical futures.

Bierbooms et al [12] explored future residential options for individuals with mental health disorders in Eindhoven. They adhered to van der Heijden’s [13] deductive scenario method. They began with a deep examination of the external environment using document analysis and stakeholder interviews. Experts from different areas, such as mental health services, housing corporations, municipal authorities, and patient advocacy associations, identified 2 key uncertainties: the availability of financial resources and the potential for effective social integration of individuals with mental health issues. These uncertainties formed the 4 scenarios that were evaluated in a stakeholder workshop. They concluded that community-based care with robust social support was the most preferable scenario, regardless of financial circumstances. The results indicated that this method is an effective tool for converting macro-level trends into actionable, real-world local policies. Consistent with earlier research, this study also concluded that stringent collaboration between health care providers and policymakers is essential.

In another study, Ludwig et al applied SA in a workshop to examine the future of academic health sciences libraries [14]. Participants identified key drivers of change and developed 4 possible scenarios for libraries: “Library, Inc.,” wherein libraries operate as profit-oriented organizations; “Evolution to Reformation,” characterized by their complete integration into clinical, research, and educational frameworks; “If Disney® Ran the Library,” emphasizing user experience and public engagement; and “Go Global,” which prioritizes international collaboration and the public accessibility of important health information. Each scenario brings distinct obstacles and, naturally, opportunities from financial perspectives and open-access policies. Likewise, they assist librarians in identifying their new roles in an increasingly digital landscape. SA empowers libraries to mitigate risks and consider their realistic opportunities while anticipating possible futures.

### Backcasting

Backcasting, often following SA or visioning, is a futures method that starts with the definition of a preferred or a plausible future state and then works backward to the present to identify the necessary steps to achieve this vision. The approach is particularly useful for recognizing potential roadblocks, necessary interventions, and policy changes required to reach the future goal. By focusing on the end goal rather than just extrapolating from actual trends, backcasting allows for the development of a structured and strategic pathway toward the envisioned future.

The process is distinctly normative, since it delineates the necessary policy actions required to attain a specified future end point from the present. The method is used in situations where a normative objective exists, although fundamentally uncertain future occurrences influence these objectives [15]. It involves identifying key stakeholders, resources, and decision points, ensuring that all of the relevant factors are considered in the implementation process [16]. An innovation champion is often designated to advance the initiative [17]. Backcasting is particularly useful in complex systems like health care, where foresight accuracy is limited and the most likely future may not align with the most desirable one.

The COVID-19 pandemic has underscored the urgent need for proactive strategies to strengthen health care systems [18]. A study used backcasting to identify actionable measures for achieving global health resilience by 2030. The study delineated a preferred future state of health system preparedness and retroactively formulated 13 strategic plans encompassing policy changes, health care system reforms, and international collaboration. By addressing key challenges such as pandemic response, health equality, and sustainable health care practices, it provided a roadmap for governments, health organizations, and international institutions to improve pandemic preparedness and global health security. This comprehensive approach demonstrates how systematic foresight might yield plausible solutions to global health challenges in the postpandemic era.

To facilitate eHealth adoption, researchers conducted focus group interviews with middle managers and staff across different health care service settings to identify roadblocks and facilitators of digital health care implementation [19]. These insights provided valuable information for understanding management challenges and guiding conversations on future-oriented decision-making. The authors highlighted the method’s potential as an invaluable instrument for structuring future decisions by reverse-engineering from a preferred health care scenario to determine necessary steps and policy changes while stepping away from the limitations of present-focused thinking. Backcasting is presented as a framework that can help address uncertainties, establish a rational basis for decision-making, and foster active stakeholder engagement in complex transformations. The research exemplifies how empirical evidence on current challenges can be used to formulate a future vision and delineate actionable strategies for its achievement.

### Technology Assessment

Health technology assessment (HTA) is a multidisciplinary process that systematically investigates the medical, social, ethical, and economic implications of a health technology. In a complex, evolving health care environment, HTA aids decision-makers in comprehending the implications for all stakeholders (patients, health care providers, payers, and policymakers) while providing an independent appraisal of the benefits and detriments of technology to guide economic decisions. HTA facilitates the integration of diverse evidence assessments to ensure the right use of technologies, safeguarding the optimal and ethical allocation of health care resources [20].

HTA types may denote the methods of data analysis, the timing or depth of the evaluation, or the particular technologies under examination. Principal types encompass primary data methods (such as clinical trials) and integrative approaches (including systematic reviews) for data synthesis, in addition to full and rapid HTA with varying degrees of urgency and financial constraints [21,22]. HTA encompasses evaluations of various categories of health technology, including drugs, medical devices, procedures, and public health programs.

One past study aimed at providing guidance on how the patient’s perspective should actively influence decisions in technological development [23]. The authors contended that it is essential to incorporate patient preferences from the outset of the design process, rather than treating their input as an afterthought. The study used HTA to systematically evaluate the clinical, economic, and societal impacts of health technologies, incorporating direct feedback from end users and considering patient-level outcomes, preferences, and values. This patient-centered approach exemplifies inclusive and transparent assessment, as it ensures that real-world perspectives guide policy and practice. It highlights that including patient viewpoints can improve the relevance, adoption, and overall success of health innovations, proving HTA’s potential to adjust to shifting expectations in health care.

This idea is particularly true when health systems need to determine the value of new and emerging technologies, such as precision medicine. Love-Koh et al [24] proposed that traditional evaluation models are insufficient for assessment due to the integration of unique patient data, including genetic, environmental, and lifestyle aspects, necessitating the development of new, adaptive frameworks. They did not offer a single, definitive way of changing the evaluation, but suggested a variety of methodological modifications in economic modeling, evidence standards, and evaluation timing. The study combined a literature review with expert interviews, allowing a comprehensive exploration of possible obstacles. Using HTA principles, the research delineates deficiencies in current HTA models and highlights the need for more flexible, responsive assessments. This article illustrates that HTA needs to continue to adapt its existing ways of working in order to remain functional in a meaningful and useful way in these innovative and rapidly changing health care settings.

However, HTA must remain systematic and evidence based, even during rapid public health crises like the COVID-19 pandemic, to support proper decision-making. Cadeddu et al [25] described a practical example from the first 12 months of the COVID-19 vaccination rollout in Italy that included HS combined with multicriterion decision analysis to guide HTA to assess vaccine candidates based on a variety of criteria, such as distribution logistics and availability. In this article, HTA demonstrated its strength in providing actionable and relevant advice even in rapidly changing times, showing itself to be a robust, evidence-based tool.

This demonstrates HTA’s critical role in guiding future research and policy development in the context of personalized medicine.

### Policy Analysis

Policy analysis is a systematic method applied to public policies to determine their efficacy, implications, and consistency with societal objectives. It entails determining policy problems, analyzing current policies or suggested solutions, and projecting their likely impacts along multiple dimensions, such as social, economic, ethical, and political aspects. By applying methods such as literature reviews, stakeholder analysis, interviews, or comparative analysis, policy analysis attempts to inform decision-makers to make strategically appropriate, plausible choices to solve complex challenges.

Policy analysis uses diverse qualitative (eg, case studies and interviews) and quantitative (eg, cost-benefit analysis, statistical modeling, and surveys) methodologies to systematically examine and assess public policies [26]. Essential steps encompass problem definition, policy option development and analysis, and solution recommendation. Methods are classified into descriptive analysis, which evaluates existing policies, and prescriptive analysis, which offers remedies and recommendations.

Another study analyzed the challenges faced by rural health care providers and illustrated how policy analysis can be used to form an effective response [27]. The authors used policy analysis to identify targeted interventions that can enhance sustainability and quality improvements, acknowledging that rural health care providers are especially vulnerable to the impacts of public policy changes due to constraints on the size of their practices and on resources. The research identifies deficiencies in current health care policies affecting rural health care providers by analyzing existing literature and policy contexts, demonstrating that policy analysis offers a systematic approach to comprehending these sociopolitical dynamics. This methodological approach delineates actionable steps for policymakers, establishing a definitive framework for improving rural health care infrastructure.

Another study used policy analysis to anticipate and guide policy change, using the issue of legalizing abortion in Indonesia as a case study [28]. Recognizing the intricacies of policy changes, the researchers applied a well-established policy process theory, the advocacy coalition framework, to analyze stakeholders’ beliefs, alliances, and influence. The analysis revealed that policy change was substantially affected by stakeholder coalitions and their foundational ideas through the examination of debates, stakeholder positions, and alliances. This demonstrates the significance of policy analysis in anticipating policy changes and informing strategic approaches to achieve desired outcomes.

### The HS Method

HS is a systematic, future-oriented method for identifying and analyzing emerging trends, innovations, or weak signals. Its objective is to facilitate policymaking, decision-making, and resource allocation in advance by addressing emerging risks, enhancing resilience to future shocks, and mitigating uncertainty [29]. It is based on the collection and analysis of information from a wide range of sources, including online databases, literature, expert panels, stakeholder engagement, and AI-based generative pretrained transformer applications to identify early warning signs of change. The key aim is to inform strategic decision-making, policy formulation, and resource allocation in advance, enabling organizations to anticipate developments and respond accordingly to future challenges or opportunities.

Nonetheless, HS can only effectively contribute to shaping the future if it transcends mere information gathering and strategic intelligence creation as a standalone approach and is instead seamlessly incorporated into the foresight process [30]. Therefore, it is argued that nowadays, HS comprises an essential element for the effective accomplishment of any futures study or foresight project [31].

The method has demonstrated its usefulness for various strategic purposes in health care, including regulatory preparation, policy change, technology prioritization, and anticipating social and ethical effects. The research group of Rodríguez-Gómez et al [32] used it to analyze how nanotechnology-enabled products in health care would possibly alter existing regulatory frameworks, suggesting that anticipatory methods could reduce delays in decision-making and better align oversight systems with regulation without hindering innovation. Similarly, Michels et al [33] analyzed the development of an international HS tool to identify medical devices at an early stage of their life cycle, showing how the tool was shaped by different perspectives, such as regulation, HTA, and resource-sharing among small countries. In both examples, HS was used to support system-level preparedness rather than prediction in a narrow sense.

Emerging technology identification and prioritization are also areas where HS has proven useful. The authors of one study described a systematic approach involving biomedical engineers to identify remote monitoring tools for national assessment. They informed their judgment using various sources, including literature, expert input, and benchmarks, to recommend actions for the UK Department of Health and Social Care [34]. A more comprehensive picture was provided by Ormstad et al [35], who compared national HS systems for medical devices, looking at differences in scope, timing, and institutional structure. They concluded that even if the practical approaches to the method might be dissimilar, their function as tools for early awareness remained unchanged.

The method can assist in discovering ethical and social issues associated with new information and communication technologies in health care and aging. Acknowledging the rapid technological development and related ethical uncertainty surrounding this subject, the authors of one study applied a structured HS method using systematic literature reviews, expert interviews, and analysis of online discussions to identify both weak and strong signals [36]. They successfully highlighted important ethical and social concerns, including stereotyping of older persons, privacy and informed consent, autonomy concerns, and apprehension around technology use.

In response to the emerging market context, Yeoman et al [37] evaluated trends that might anticipate how wellness and aging would shape behaviors and expectations for future consumers and services. In these cases, HS was beneficial for foresight, beyond the scope of technical systems, and helped to direct attention toward changes in society that are closely tied to health care needs.

### The Delphi Method

The Delphi method is a structured technique used to achieve expert consensus on complex topics. It involves a panel of relevant experts who typically participate anonymously in 2 or more rounds of surveys to gather their opinions on the subject matter. Following each round, the survey responses are summarized by the facilitator and shared with the participants. Based on this collective feedback, the panelists can reassess their previous answers, leading them to converge on a common answer over subsequent survey rounds. This process is repeated until the predefined stopping criterion is reached, such as the achievement of consensus. The Delphi method is a structured conversation process that elucidates the rationale behind extreme viewpoints and facilitates feedback.

The main variants of the Delphi method encompass classical Delphi, used for trend forecasting via several rounds of anonymous input; policy Delphi, intended to formulate strategies for tackling specific issues; and decision Delphi, which seeks to enhance decision-making results [38]. Additional variations encompass the ranking-type Delphi, used for issue prioritization, and the disagreement Delphi, which investigates subjects lacking initial consensus [39].

To investigate the required skills and attitudes in the digital health curriculum for medical students, the authors of one study conducted a scoping review and Delphi method study [40]. The scoping review was based on the research question “What knowledge, skills and attitudes (KSAs) within digital health are essential for future doctors?” This served as the basis to identify relevant topics for inclusion in the Delphi questionnaire. The participatory expert panel included 18 individuals who worked professionally in digital health research or implementation or were actively involved in developing medical education curricula with knowledge of digital health. After completing 2 questionnaire rounds, the expert panel identified 40 digital health topics, including a range of knowledge, skills, and attitudes, to be taught during medical school. The findings can provide medical educators with adequate insights for developing future digital health curricula.

The method can also be used to obtain a consensus regarding the required competencies for medical graduates to use AI technologies in practice [41]. One Delphi study analyzed this in 3 rounds via online survey questionnaires. This was completed by an interdisciplinary panel of 60 experts who expressed strong agreement on 23 items. These items served as the basis for the list of competencies that medical graduates require in order to be ready for AI in medical practice. This insight can be used to inform the development of medical curricula to integrate AI competencies.

Another Delphi study was conducted to derive a likely scenario for the use of voice-controlled intelligent personal assistants in health care within the next 5 years [42]. It featured an international, interdisciplinary panel of experts. The questionnaires involved 4 thematic sections (technology, consumer acceptance, potential use cases, and privacy and data protection regulations), and 27 participants completed the second round of questionnaires. There was a high level of consensus for the potential of such technologies to assist older people, for their widespread use across various health care domains, and for their use not as a replacement for medical staff but rather as a supportive tool.

A 3-round Delphi study in which 33 experts with diverse roles took part helped better understand the perceptions of Finnish experts regarding future hospital management and leadership in the year 2030 [43]. The two main categories that emerged from the experts’ perceptions were (1) management and leadership orientation and (2) future organization. In relation to management and leadership orientation, the experts perceived patient-centeredness, clinical dominance, professional divisions, and management career options as key factors. Their perceptions relating to future management and leadership organization involved shared, pair, team, and individual leadership. The insights derived from these findings can be used to plan, develop, and update management and leadership training as well as management practices in health care institutions.

## Discussion

### Overview

The case studies presented in this paper demonstrate that the analysis of future directions, scenarios, and visions can be grounded in systematic and validated methods that have proven their efficacy in other industries over the last decades. The use of futures methods in medical and health care settings, as well as in policy- and decision-making, is long overdue.

**Figure 3:**
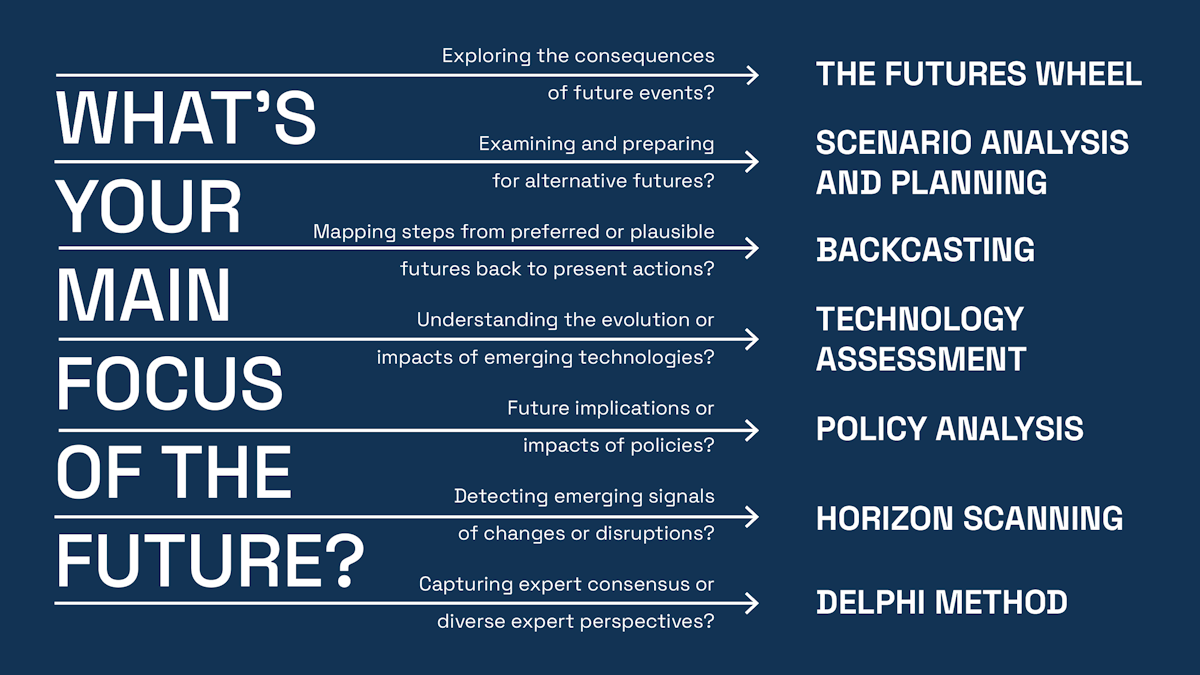
A summary of futures methods and for what purposes each method could be a good fit.

Given the diverse nature of futures methods, it is important to recognize that each is designed to address different aspects of foresight, from exploring uncertainty to building strategic visions, which is crucial for their effective use in health care contexts. The FW is used to determine the rippling effects and consequences of future milestones or events. SA and SP facilitate the development of strategies around future visions. Subsequently, backcasting facilitates translating those strategies into actionable steps today. HTA enables the effective adoption of advanced technology, while policy analysis supports policymakers in enhancing regulation. HS can be used to identify weak signals that might become trends later to anticipate major changes in a given field. The Delphi method highlights the importance of equal opinions about the future of fields of interest (Figure 3).

Futurists frequently use methodological combinations to deepen their research and improve its effectiveness. Methodological combinations can merge the benefits of distinct techniques, yielding more reliable outcomes than when using the methods separately [44]. The integration of methods is especially advantageous, as reliance on a singular method rarely provides a complete picture of the future. It advocates for the use of various methods tailored to certain contexts, facilitating a comprehensive comprehension of their advantages and limitations, along with their potential applications [45].

Moreover, futures methods inherently encourage interdisciplinary collaboration by creating structured frameworks that require diverse perspectives to envision, analyze, and prepare for multiple possible futures. These approaches shift the focus from siloed expertise to collective intelligence, making them particularly powerful in complex domains like health care, where challenges span clinical practice, policy, technology, ethics, and human behavior.

By design, methods such as SP, the FW, and backcasting invite contributions from professionals with different backgrounds—physicians, technologists, economists, designers, patients, ethicists, and policymakers. This inclusive engagement ensures that future-oriented strategies are not only scientifically grounded but also socially relevant, ethically sound, and practically feasible.

As medicine increasingly intersects with fields like data science, robotics, environmental studies, and behavioral economics, futures methods offer an essential toolset for coordinating these domains. Their adoption in health care can serve as a model for how complex, human-centered industries can evolve through collaborative foresight.

### Challenges and Pitfalls in Using Futures Methods

Futures methods are useful tools for structured anticipation but using them includes potential pitfalls that require thoughtful navigation. One main issue comes from the future’s inherent nature: it is uncertain, and our ability to conceive of alternatives to the present is limited by our paradigms, producing a potential for presentism and narrow thinking [46]. This clearly demonstrates the necessity of remaining reflexive and self-critical when practicing foresight exercises.

Choosing the appropriate method is also not always a straightforward task. There is no normative standard for which tool to use in one situation or another, and presumably, most practitioners’ choice is based on personal preference rather than methodological appropriateness. Additionally, foresight generates prospective future scenarios that cannot be tested in the present; it is difficult to verify the findings. This concern should not diminish the value of futures methods; rather, it should emphasize careful planning, transparent reasoning, and the idea that foresight is a tool to prepare and not to predict.

Therefore, the merit of futures studies lies not in the precision of their forecasts. The objective of futures studies is not to predict the future, which is unattainable, but to facilitate improved decision-making in the present by prompting the exploration of opportunities and risks and contemplating strategies to address them. Although the future is inherently unpredictable, the judicious application of theoretical and methodological resources from futures studies enables constructive foresight and the potential to influence the future toward favorable outcomes.

Beyond methodological challenges, the practical implementation of futures methods faces additional barriers rooted in human and organizational behavior. Many of these exercises largely rely upon the judgment of experts or group facilitation; they could be subject to cognitive biases, such as confirmation bias, groupthink, and overt reliance on known assumptions. These subjective lenses can subtly affect method choices, as well as the outcomes that are emphasized. A further pitfall is overconfidence in certain scenario outputs, especially if one becomes too prominent and taken as a projection of what is expected rather than just one possible future. This can result in a limitation on adaptive thinking and can be too restrictive for strategy formation. Lastly, even well-designed methods can exhibit barriers to translating insights into action. Institutional barriers, including short-term planning cycles, limited futures literacy, and lack of leadership support, can hinder taking futures sensing into policies or practices. Avoiding some of these barriers requires not only methodological awareness but organizational preparedness to address uncertainty in genuine and sustained ways.

### Call to Action: Encouraging Health Care Institutions To Apply Futures Thinking

The rapid pace of evolution of the health care landscape in the 21st century, from technological progress to climate change, presents unpredictable challenges that necessitate more than hindsight. As we emphasized in our benchmark study, there is an imperative need to integrate futures methods within this landscape, ideally through a distinctive “medical futures studies” subdiscipline, to better equip health care stakeholders to address forthcoming challenges in the digital health era.

Despite the clear benefits of such methods, adoption in the health care field remains low; as such, we encourage health care institutions to adopt and apply futures thinking within their routine practices. To that end, we issue a call to action for widespread adoption of medical futures methods and propose the following key actions (Table 1).

- Form a dedicated medical futures team by formally appointing a team of futurists, or at least future-oriented people, who oversee the integration of futures thinking within the institution.
- Improve futures literacy by incorporating the fundamentals of medical futures methods into staff training and upskilling to develop an anticipatory and proactive mindset.
- Ensure policy integration and embed futures thinking in the institution’s major projects and decision-making.
- Build research networks based on the knowledge base by conducting academic research involving medical futures methods within the institution and with collaborators, and share the findings to help develop field-specific frameworks and best practices.
- Practice futures methods and exercise simpler ones such as SA or the FW in brainstorming sessions without the need for a practical outcome.

**Table 1:** Calls to action.

Action item	Overview
Dedicated medical futures team	Oversee integration of futures thinking
Futures literacy	Training and upskilling in medical futures methods and mindset
Policy integration	Futures thinking embedded within key projects and decision-making
Research networks	Build on and contribute to the knowledge base
Practice futures methods	Exercise simpler futures methods in brainstorming sessions

Through such actions, health care and medical institutions can effectively adopt a proactive mindset, which is crucial in the digital health era. By embracing futures thinking at every level, organizations can guide innovative practices and prepare for unforeseen obstacles, leading to a resilient and future-ready health care landscape.

It is important to point out that one of the underappreciated advantages of futures methods is their accessibility and adaptability for low-resource settings. Many techniques, such as the FW, backcasting, or basic SA, require minimal technological infrastructure and can be facilitated with just a pen and paper or simple digital tools. Because these methods prioritize structured thinking, collaborative dialogue, and creative ideation rather than costly data or proprietary software, they can empower local stakeholders, from clinicians and community health workers to patients, to cocreate context-sensitive strategies. In low- and middle-income regions, where health systems face chronic shortages of staff, infrastructure, and funding, futures thinking offers a cost-effective and scalable approach to anticipate emerging challenges and identify innovative, locally grounded solutions.

AI, especially generative AI in the form of large language models (LLMs), also acts as a facilitator in the process. LLMs can support HS by sifting through vast volumes of data to identify weak signals or emerging patterns that human analysts might overlook. They can also summarize stakeholder interviews or generate possible futures narratives based on diverse inputs. In general, LLMs can help democratize foresight by lowering the technical barriers for nonexperts to engage in futures exercises. However, this potential must be balanced with ethical oversight to ensure transparency, avoid algorithmic bias, and maintain human agency in shaping desirable futures.

### Conclusions

In an era defined by rapid technological disruption, shifting demographics, and unpredictable global events, the ability to anticipate and prepare for change is essential. Futures methods provide health care organizations with the structured tools and collaborative frameworks needed to move from reactive crisis management to proactive, strategic thinking.

By embracing these methods, every health care organization—regardless of size, geography, or resources—can build resilience, drive innovation, and ensure that decisions made today are aligned with the needs of tomorrow.

The integration of futures thinking should become a core competency in health care leadership, planning, and education, not just as a means of survival, but as a foundation for shaping a more equitable, adaptable, and future-ready health system.

## Abbreviations

AI: artificial intelligence
FW: futures wheel
HS: horizon scanning
HTA: health technology assessment
LLM: large language model
SA: scenario analysis
SP: scenario planning
STEEP: social, technological, economic, environmental, and political
